# A cross‐sectional study comparing men who have sex with men and inject drugs and people who inject drugs who are men and have sex with men in San Francisco: Implications for HIV and hepatitis C virus prevention

**DOI:** 10.1002/hsr2.704

**Published:** 2022-07-12

**Authors:** Adelina Artenie, Shelley N. Facente, Sheena Patel, Jack Stone, Jennifer Hecht, Perry Rhodes, Willi McFarland, Erin Wilson, Peter Vickerman, Meghan D. Morris

**Affiliations:** ^1^ Bristol Medical School, Population Health Sciences Institute University of Bristol Bristol UK; ^2^ Division of Epidemiology and Biostatistics, School of Public Health University of California Berkeley Berkeley California USA; ^3^ Facente Consulting Richmond California USA; ^4^ Department of Epidemiology and Biostatistics University of California San Francisco San Francisco California USA; ^5^ San Francisco AIDS Foundation San Francisco California USA; ^6^ Springboard HealthLab Berkeley California USA; ^7^ University of California San Francisco Alliance Health Project San Francisco California USA; ^8^ San Francisco Department of Public Health San Francisco California USA

**Keywords:** HCV, HIV, MSM, MSM‐IDU, PWID

## 1 INTRODUCTION

People who inject drugs (PWID) and men who have sex with men (MSM) carry a high burden of HIV and hepatitis C (HCV) and represent key populations for eliminating these viral infections.^1^, ^2^ Previous studies illustrated that MSM who inject drugs (MSM‐IDU) and male PWID who engage in sex with other men (PWID‐MSM) have greater injecting or sexual risk behaviors and HCV and HIV prevalence than other MSM and PWID, respectively.^3^, ^4^, ^5^, ^6^ However, it remains unclear how MSM‐IDU and PWID‐MSM compare to each other. People with dual risk behaviors are typically omitted from HIV/HCV programs and elimination plans or treated as a single generic risk category, with most interventions being geared towards either PWID or MSM.^1^, ^2^

Our aim was to characterize similarities and differences between MSM‐IDU (i.e., men reached through affiliation with MSM) and PWID‐MSM (i.e., men reached through affiliation with PWID) in San Francisco by comparing sociodemographic, drug use and sexual risk behaviors, and service access. We also compared the characteristics of MSM‐IDU to MSM non‐IDU and PWID‐MSM to male PWID non‐MSM to gain a broader understanding of these groups.

## 2 METHODS

We used data from the National HIV Behavioral Surveillance surveys among MSM (2017) and PWID (2018) in San Francisco. MSM were recruited using time‐location sampling at MSM venues and were eligible for enrollment if ≥18 years old and either identified as MSM or had past‐year sex with another man.^7^ PWID were recruited through respondent‐driven sampling and were eligible if ≥18 years and reported past‐year injection drug use.^7^ In both surveys, participants provided informed consent, completed the same core questionnaire, and were tested for HIV (both surveys) and HCV antibody status (PWID survey).^7^ Both studies were approved by the institutional review boards of the Centers for Disease Control and Prevention.

We restricted the PWID sample to male participants. Those who reported past‐year sex with a man were categorized as PWID‐MSM. Among the MSM sample, we categorized those who reported past‐year injecting drug use as MSM‐IDU. We categorized the remaining groups as PWID non‐MSM and MSM non‐IDU. We explored differences between groups using Pearson's *χ* ^2^ or Fisher's exact tests when expected cell counts were ≤5 for categorical variables and Mann–Whitney *U* tests for continuous variables. Among PWID, we presented sample proportions unadjusted for respondent‐driven sampling since evidence suggest they may be more representative compared to adjusted estimates.^8^ All tests were two‐sided (*α *= 0.05) and conducted using SAS v.9.4.

## 3 RESULTS

Of 504 participants completing the MSM survey, 31 (6.2%) were classified as MSM‐IDU. Of 311 male participants completing the PWID survey, 59 (19.0%) were classified as PWID‐MSM (Table 1).

**Table 1:** Sociodemographic, injection drug use and sexual behaviors, and access to services among men who have sex with men and inject drugs (MSM‐IDU) or who do not inject drugs (MSM non‐IDU) and men who inject drugs and have sex with other men (PWID‐MSM) or who do not have sex with other men (PWID non‐MSM)

Characteristic	MSM‐IDU (*n* = 31)	PWID‐MSM (*N *= 59)	*p* *	MSM non‐IDU (*n* = 473)	*p* **	PWID non‐MSM (*n* = 252)	*p* ***
*Sociodemographic factors*							
Age			0.05		0.17		0.14
18–39	20 (64.5%)	25 (42.4%)		245 (51.8%)		81 (32.1%)	
40+	11 (35.5%)	34 (57.6%)		228 (48.2%)		171 (67.9%)	
Race/ethnicity			<0.01		0.58		0.34
White	20 (64.5%)	23 (39.0%)		238 (50.3%)		122 (48.4%)	
Black/African–American	1 (3.2%)	12 (20.3%)		26 (5.5%)		52 (20.6%)	
Hispanic or Latino/a/x	6 (19.4%)	5 (8.5%)		98 (20.7%)		26 (10.3%)	
Multiple	3 (9.7%)	17 (28.8%)		56 (11.8%)		43 (17.1%)	
Other	1 (3.3%)	2 (3.4%)		55 (11.6%)		9 (3.6%)	
Sexual identity			<0.01		<0.01		<0.01
Heterosexual	4 (12.9%)	9 (15.3%)		5 (1.1%)		236 (93.7%)	
Gay	22 (71.0%)	23 (39.0%)		429 (90.7%)		2 (0.8%)	
Bisexual	5 (16.1%)	27 (45.8%)		39 (8.3%)		14 (5.6%)	
Highest level of education completed			0.09		<0.01		0.21
High school or less	11 (35.5%)	32 (54.2%)		53 (11.2%)		159 (63.1%)	
Some college, Bachelor's degree, and above	20 (64.5%)	27 (45.8%)		420 (88.8%)		93 (36.9%)	
Current employment status			<0.01		0.06		0.97
Employed	17 (54.8%)	5 (8.5%)		341 (72.1%)		19 (7.5%)	
Unable to work for health reasons	3 (9.7%)	21 (35.6%)		16 (3.4%)		89 (35.3%)	
Not employed	11 (35.5%)	33 (55.9%)		116 (24.5%)		144 (57.1%)	
Household income			<0.01		<0.01		0.83
US$ 0–24,999	12 (38.7%)	49 (83.1%)		78 (16.5%)		217 (86.1%)	
US$ 25,000–49,999	5 (16.1%)	7 (11.9%)		92 (19.5%)		24 (9.5%)	
≥US$ 50,000	14 (45.2%)	3 (5.1%)		303 (64.1%)		11 (4.4%)	
Currently homeless	8 (25.8%)	39 (66.1%)	<0.01	11 (2.3%)	<0.01	206 (81.7%)	<0.01
Ever held in detention, jail or prison >24 h	14 (45.2%)	53 (89.8%)	<0.01	73 (15.4%)	<0.01	244 (96.8%)	0.03
*Injection drug use behaviors*							
Age at first injection (Median, IQR)	30 (23‐39)	22 (16‐30)	<0.01	n/a		20 (16‐26)	0.30
Drug most often injected, past 12 months			0.53				<0.01
Meth/amphetamine	20 (64.5%)	32 (54.2%)		n/a		46 (18.3%)	
Heroin	4 (12.9%)	13 (22.0%)		n/a		148 (58.7%)	
Other	7 (22.6%)	14 (23.7%)		n/a		58 (23.0%)	
Drugs injected, past 12 months							
Speedball	1 (3.2%)	17 (28.8%)	<0.01	n/a		137 (54.4%)	<0.01
Heroin	6 (19.4%)	28 (47.5%)	0.01	n/a		216 (85.7%)	<0.01
Powder cocaine	1 (3.2%)	10 (16.9%)	0.09	n/a		82 (32.5%)	0.02
Crack cocaine	1 (3.2%)	4 (6.8%)	0.66	n/a		40 (15.9%)	0.07
Methamphetamine	21 (67.7%)	54 (91.5%)	<0.01	n/a		170 (67.5%)	<0.01
Painkillers^a^	9 (29.0%)	9 (15.3%)	0.17	n/a		68 (27.0%)	0.06
Injected ≥2 different drug types, past 12 months	8 (25.8%)	35 (59.3%)	<0.01	n/a		203 (80.6%)	<0.01
Daily injection, past 12 months	9 (29.0%)	38 (64.4%)	<0.01	n/a		204 (81.0%)	<0.01
Used injection equipment previously used by someone else, past 12 months	8 (25.8%)	24 (40.7%)	0.16	n/a		102 (40.5%)	0.98
Overdose, past 12 months^b^	1 (9.1%)	11 (30.6%)	0.15	0	0.30	64 (28.1%)	0.76
*Sexual behaviors*							
Number of male sexual partners, past 12 months (Median, IQR)	10 (6‐20)	3 (1‐10)	<0.01	7 (2‐20)	0.38	n/a	
Condomless anal intercourse, past 12 months	29 (93.6%)	37 (62.7%)	<0.01	381 (80.6%)	0.07	n/a	
Received money or drugs from a man to have sex, past 12 months	11 (35.5%)	30 (50.9%)	0.16	26 (5.5%)	<0.01	n/a	
Had a female sex partner, past 12 months	7 (22.6%)	30 (50.9%)	<0.01	31 (6.6%)	<0.01	183 (72.6%)	<0.01
*Testing and use of services*							
Drug treatment, past 12 months	6 (19.4%)	18 (30.5%)	0.26	22 (4.7%)	<0.01	70 (27.8%)	0.68
MOUD, past 12 months^b^	3 (27.3%)	16 (44.4%)	0.31	0	0.02	142 (62.3%)	0.04
Obtained sterile needles from a SSP, past 12 months	11 (35.5%)	51 (86.4%)	<0.01	n/a		239 (94.8%)	0.04
Obtained sterile syringes from a pharmacy, past 12 months	17 (54.8%)	29 (49.2%)	0.61	n/a		92 (36.5%)	0.07
Tested for HIV, past 12 months^b^	19 (90.5%)	30 (75.0%)	0.19	304 (79.0%)	0.21	159 (66.8%)	0.30
Tested for HCV, past 12 months	28 (90.3%)	36 (61.0%)	<0.01	258 (54.6%)	<0.01	187 (74.2%)	0.04
PreP awareness^c^	18 (85.7%)	29 (72.5%)	0.24	373 (96.9%)	0.04	115 (48.3%)	<0.01
PreP use, past 12 months^c^	9 (42.9%)	6 (15.0%)	0.02	171 (44.4%)	0.89	1 (0.4%)	<0.01
HIV‐positive	10 (32.3%)	23 (39.0%)	0.53	87 (18.4%)	0.06	15 (5.9%)	<0.01
HCV antibody positive	n/av	42 (71.2%)		n/av		200 (79.4%)	0.17
Currently receives antiretroviral therapy^d^	8 (80.0%)	18 (78.3%)	0.99	81 (93.1%)	0.19	11 (73.3%)	0.99
Ever received treatment for hepatitis C^e^	n/a	15 (35.7%)		n/a		61 (30.5%)	0.58

*Note*: *p*‐values were derived based on Pearson's *χ* ^2^ test or, alternatively, Fisher's exact test when expected cell counts were ≤5 for categorical variables, and Mann–Whitney *U* test for continuous variables

Abbreviations: HCV, hepatitis C virus; HIV, human immunodeficiency virus; IQR, interquartile range; MOUD, medication for opioid use disorder; n/a, not applicable; n/av, not available; PrEP,pre‐exposure prophylaxis; SSP, syringe service program.

^a^ This category includes oxycontin, dilaudid, morphine, percocet, or demerol.

^b^ Data available among participants who used opioids in the previous year only (*n* = 11, 36, 26, and 228 among MSM‐IDU, PWID‐MSM, MSM non‐IDU, and PWID non‐MSM, respectively); MOUD refers to treatment with methadone or buprenorphine.

^c^ Data presented among participants who report being HIV‐negative (*n* = 21, 40, 385, and 238 among MSM‐IDU, PWID‐MSM, MSM non‐IDU, and PWID non‐MSM, respectively).

^d^ Data presented among participants who tested HIV‐positive, as presented in the table.

^e^ Data presented among participants who tested HCV antibody positive, as presented in the table.

* *p*‐value comparing MSM‐IDU to PWID‐MSM

** *p*‐value comparing MSM‐IDU to MSM non‐IDU

*** *p*‐value comparing PWID‐MSM to PWID non‐MSM.

### 3.1 Comparing MSM‐IDU and PWID‐MSM

MSM‐IDU and PWID‐MSM were different across numerous sociodemographic measures. PWID‐MSM were older than MSM‐IDU (57.6% vs. 35.5% were ≥40 years), more racially/ethnically diverse (61.0% vs. 35.5% identified as nonwhite), and more were bisexual (45.8% vs. 16.1%). More PWID‐MSM reported a household annual income of <$25,000, current homelessness, and prior incarceration.

Although a similar proportion of MSM‐IDU (64.5%) and PWID‐MSM (54.2%) indicated methamphetamine as the drug most often injected, other injection drug use and sexual behaviors differed. Compared to MSM‐IDU, PWID‐MSM began injecting drugs earlier (median age: 22 vs. 30 years), more injected ≥2 different drugs (59.3% vs. 25.8%), and injected daily (64.4% vs. 29.0%). Conversely, PWID‐MSM reported fewer male sexual partners (median: 3 vs. 10), less condomless anal sex (62.7% vs. 93.6%), and more reported a female sex partner (50.9% vs. 22.6%).

Service use also differed across the two groups. More PWID‐MSM sought sterile syringes from a syringe service program than MSM‐IDU (86.4% vs. 35.5%). Conversely, more MSM‐IDU reported using pre‐exposure prophylaxis (PrEP) (42.9% vs. 15.0%) and having been HCV‐tested (90.3% vs. 61.0%) than PWID‐MSM. HIV prevalence was similarly high for both MSM‐IDU (32.3%) and PWID‐MSM (39.0%). We also noted nonstatistically significant differences between MSM‐IDU and PWID‐MSM on receipt of medications for opioid use disorder, sharing practices, overdose, and HIV testing.

### 3.2 Comparing MSM‐IDU and MSM non‐IDU

Several characteristics differed between MSM‐IDU and MSM non‐IDU. For example, a larger proportion of MSM‐IDU reported lower education (35.5% vs. 11.2%) and income (38.7% vs. 16.5%), current homelessness (25.8% vs. 2.3%), and prior incarceration (45.2% vs. 15.4%). More MSM‐IDU received money or drugs in exchange for sex with a man (35.5% vs. 5.5%), had a female sex partner (22.6% vs. 6.6%), and were HIV‐positive (32.3% vs. 18.4%) relative to MSM non‐IDU.

### 3.3 Comparing PWID‐MSM and PWID non‐MSM

PWID‐MSM and PWID non‐MSM were comparable on several sociodemographic measures. However, more PWID‐MSM reported methamphetamine as their primary drug injected (54.2% vs. 18.3%) while fewer reported heroin (22.0% vs. 58.7%) compared to PWID non‐MSM. A larger proportion of PWID‐MSM were aware (72.5% vs. 48.3%) and had used PrEP (15.0% vs. 0.4%). HIV prevalence was higher among PWID‐MSM compared to PWID non‐MSM (39.0% vs. 5.9%); no difference was found for HCV prevalence (71.2% vs. 79.4%).

## 4 DISCUSSION

Overall, compared to MSM‐IDU, PWID‐MSM presented greater socioeconomic disadvantage and reported heavier injecting drug use but lower sexual risk practices. While MSM‐IDU was more engaged in MSM‐oriented prevention programs like PrEP, PWID‐MSM was more engaged with syringe services programs, which primarily target PWID. Although the strength of these findings is limited by small sample sizes, taken together, our results suggest that MSM‐IDU and PWID‐MSM represent distinct populations that are present in different social spaces and should not be conflated with one another. More broadly, these findings suggest that harm reduction and healthcare settings catering to MSM and PWID, like syringe services programs and sexual health clinics, should adapt to reflect the complexity of risk practices and needs of these groups and provide a wider range of HIV/HCV prevention services.

The extent to which people who engage in both injecting‐ and sexual‐risk behaviors were included in the PWID‐ or MSM‐focused studies could reflect the primary behavior which takes precedence in day‐to‐day life. In a qualitative study focused on people with dual risk behaviors, some participants reported engaging in male‐to‐male sex work to sustain injection drug use, whereas others indicated that injection drug use was used to enhance male‐to‐male sex.^9^ Varying motivations and levels of priority assigned to injecting and sexual practices have been reported in other studies^10^ and explain why some individuals may not identify as MSM or PWID.^9^ A better understanding of the reasons motivating these practices could increase the extent to which HCV and HIV prevention programs engage and help reduce risk behaviors among these populations.

We also noted important differences between MSM‐IDU and MSM non‐IDU and PWID‐MSM and PWID non‐MSM, respectively. One‐third of MSM‐IDU indicated receiving money or drugs from a man to have sex, whereas few (5.5%) MSM non‐IDU indicated this practice. While few PWID‐MSM indicated heroin as the most injected drug, over half of PWID non‐MSM did so. Across both MSM and PWID, HIV prevalence was higher among the dual risk groups. Collectively, these distinctions emphasize the importance of providing access to combined sexual health and harm reduction messages rather than targeting specific risk behaviors.

In conclusion, our study suggests that MSM‐IDU and PWID‐MSM have distinct demographic, risk behavior, and healthcare access profiles. Given ongoing calls to broaden access to HCV and HIV interventions among PWID and MSM to reach 2030 elimination goals, findings indicate a need to provide access to a greater range of services to both populations.

## AUTHOR CONTRIBUTIONS

**Adelina Artenie, Shelley N. Facente, Peter Vickerman, and Meghan D. Morris**: Conceptualization. **Adelina Artenie and Sheena Patel**: Data curation. **Adelina Artenie**: Formal analysis. **Adelina Artenie and Meghan D. Morris**: Funding acquisition. **Adelina Artenie and Shelley N. Facente**: Writing – original draft. **Adelina Artenie, Shelley N. Facente, Jack Stone, Jennifer Hecht, Perry Rhodes III, Willi McFarland, Erin Wilson, Peter Vickerman, and Meghan D. Morris**: Writing – review and editing. Adelina Artenie and Meghan D. Morris had full access to all of the data in this study and take complete responsibility for the integrity of the data and the accuracy of the data analysis. All authors have read and approved the final version of the manuscript.

## CONFLICTS OF INTEREST

Peter Vickerman received an unrestricted research grant from Gilead that is not related to this study. Meghan D. Morris received investigator‐sponsored research funding from Gilead Sciences for research not related to this study. Shelley N. Facente acknowledges consulting support from Gilead Sciences and from End Hep C SF; neither is related to this study. All other authors have no conflict of interest.

## TRANSPARENCY STATEMENT

Adelina Artenie and Meghan D. Morris affirm that this manuscript is an honest, accurate, and transparent account of the study being reported; that no important aspects of the study have been omitted; and that any discrepancies from the study as planned have been explained.

## Data Availability

The data that support the findings of this study are available from the National HIV Behavioral Surveillance study. Restrictions apply to the availability of these data, which were used under license for this study. Data may be available from Meghan D. Morris with the permission of the National HIV Behavioral Surveillance study.
